# A descriptive analysis of substance use screening among youth involved in the legal system in eight counties

**DOI:** 10.1186/s13722-025-00609-3

**Published:** 2025-10-21

**Authors:** Lauren O’Reilly, Allyson Dir, Katherine Schwartz, Steven Brown, Fangqian Ouyang, Patrick Monahan, Zachary Adams, Tamika Zapolski, Leslie Hulvershorn, Matthew Aalsma

**Affiliations:** 1 Indiana University School of Medicine, 410 W 10th Street, Indianapolis, IN 46202 USA; 2 Indiana University School of Medicine, Goodman Hall 355 W 16th Street Suite 4800, Indianapolis, IN 46202 USA

**Keywords:** Youth legal system, Youth, Adolescent, Juvenile justice, Substance use, Substance use screening, CRAFFT, Drug test

## Abstract

**Background:**

Substance use disorders (SUDs) are more prevalent among youth involved in the legal system (YILS) compared to non-system-involved peers. Legal system involvement can serve as a gateway to health services, presenting an opportunity to identify YILS in need of SUD intervention. Here, aims included: (1) describe YILS patterns of substance use based on drug testing (e.g., urine) versus a self-report risk screener, (2) characterize how screening practices and results differ based on YILS demographic characteristics, and (3) compare how positive screening informs referrals to behavioral health treatment by screening type.

**Methods:**

Administrative records were collected for all youth, ages 11–17, arrested in one of eight Indiana counties during 2019–2023 (*N* = 1,197 youth). Variables included youth demographics, most severe alleged charge at arrest, drug test results, self-report substance use screen results, and youth referrals to behavioral health services by probation officers. Prevalence of screening receipt by type, positive screens, and substance detected in drug tests are reported. Chi-square tests were conducted between demographic variables and drug test and self-report screener receipt, and positive screen per screening type. Logistic regression models were used to determine the association between positive screen and referral to services; models were compared using the *c*-statistic.

**Results:**

Of sampled youth, 26.7% received a drug test, and 58.0% a self-report screener; of those, 54.4% and 42.0% screened positive, respectively. Cannabinoids (84.5%) and alcohol (28.2%) were detected most often. Youth whose most severe alleged charge was drug-related were more likely to receive a drug test, to receive a self-report screener, and screen positive. Positive drug test (OR = 8.48 95% CI = 6.06–11.87) and self-report screen (OR = 11.55, 95% CI = 8.33–16.01) were associated with behavioral health referral; the *c*-statistic was slightly higher for self-report screener (0.77) than for drug test (0.74).

**Conclusions:**

Probation officers may be utilizing self-report screener results to make referral recommendations differently than drug test results. For less resourced counties that do not administer drug tests, self-report screeners may be particularly helpful in triaging youth to other services that routinely monitor substance use. Future research is needed to identify the optimal frequency of substance use screening to inform service referrals.

**Trial registration:**

The study was registered through ClinicalTrials.gov on 7/30/2020 (ID NCT04499079).

**Supplementary Information:**

The online version contains supplementary material available at 10.1186/s13722-025-00609-3.

## Background

Involvement in the youth legal system provides an ideal opportunity to identify youth (< 18 years old) in need of substance use intervention and other behavioral health services. Substance use disorders (SUDs) are more prevalent among youth involved in the legal system (YILS) compared to their peers not involved in the system. The lifetime prevalence rate of SUD among YILS is 62% compared to 6–9% among the general population [1–3]. Among youth arrested or otherwise referred to juvenile probation intake, the prevalence of any SUD is 25% [4]. Substance use during adolescence is a risk factor for criminal recidivism [5] and SUD is the most prevalent psychiatric disorder persisting into adulthood among previously detained youth [6]. Therefore, identifying and addressing SUDs among YILS is an important public health issue.

In addition to high rates of SUD among YILS, the youth legal system often acts as a gateway to community services [7]. The US Department of Justice and the Office of Juvenile Justice and Delinquency Prevention has recommended that all youth be screened for mental health and SUD, ideally at their first legal system contact [8]. Despite the recommendation, no universal screens for behavioral health treatment needs (including substance use) have been implemented at scale within the youth legal system. While adolescent substance use is associated with subsequent SUD [9], we use the term “substance use” through the remainder of the introduction as past research on screening more frequently identifies the presence of substance use rather than SUD.

### Screening for and identifying substance use service needs

Methods of identifying youth behavioral health service needs vary across jurisdictions, and the decisions of whether to use screenings and how to act upon results are often left to the discretion of system personnel [10, 11]. For instance, information gleaned through interviews with youth and their families, as well as a review of a youth’s legal case history, might provide insight into service need. Drug testing (i.e., testing urine or saliva samples for the presence of substances) is routine in both the adult and youth legal system and provides a potentially more objective method of detecting and monitoring substance use during system involvement [12]. In addition to drug tests, some youth legal agencies have utilized self-report behavioral health risk screening tools to identify substance use service needs. Results from JJ-TRIALS, a 36-site hybrid implementation study aimed to improve youth connection to behavioral health services, found that 72% of sites used at least one evidence-based self-report substance use screening measure and 47% routinely used drug tests [9]. While drug testing is a staple practice in the legal system, drug tests only indicate recent substance use not *problem* use (i.e., use associated with potential negative consequences such as using while driving or using more than intended, but not a confirmed SUD) or SUD. In contrast, self-report substance use screens may better identify problem use warranting a need for treatment in a more efficient way than interviews or case reviews.

Data from studies utilizing drug testing and self-report substance use screening have been used to estimate the prevalence of substance use among YILS [6, 13]. Yet, minimal research has examined whether self-report substance use screening tools are being administered consistently [14] and to whom. There is some, but limited, evidence for differences in drug test or screening administration. Past research on demographic differences among YILS has predominantly focused on system involvement [15], substance use [16], or referral to services [17, 18]. For racial differences, results from one Midwestern jurisdiction found differences in youth who received drug tests; male youth, those with violent offenses, and younger youth were more likely to be tested [19]. Another study found no relationship between sex, race, nor ethnicity and *positive* substance use screenings [14]. For geographic differences, research found that robustness of substance use screening varies by urbanicity, with urban agencies adopting more thorough screening practices (i.e., substance use screeners and drug tests) [20]. Additional research is needed to better understand patterns of drug testing and substance use screening, as well as related demographic differences, among YILS.

### Screening to inform decision-making

Research is also needed to learn about potential patterns in decision-making following screening and testing and, specifically, how drug testing and substance use screening are used to determine need for behavioral health treatment or other court-ordered services. As mentioned above, while research has identified racial disparities in referrals to behavioral health treatment [18, 21], less is understood regarding the process or decision-making for referrals, such as use of screening instruments. In a study comparing the utility of different validated screening instruments on treatment referral among YILS, findings revealed low rates of treatment referral regardless of which tool was used [14]. A recent study by Clifton and colleagues (2024) conducted a descriptive study in one midwestern urban county of drug screening given the variability in drug screening administration. We aim to extend this work by exploring patterns of drug testing (and substances detected), substance use screening, and treatment referrals across numerous jurisdictions to better understand how to improve substance use-related procedures in the youth legal system.

### Current study

The current study provides a descriptive examination of patterns of drug testing and screening for problem use and subsequent referral to behavioral health services across eight jurisdictions immediately following the implementation of a self-report screening tool in these counties. The purpose of the manuscript is three-fold. First, we aim to examine patterns of substance use screening (and detected use) among a large sample of YILS spanning eight counties in Indiana. Second, we aim to understand who receives and screens positive on substance use screening and drug testing by determining potential differences across demographic groups. Third, from a systems perspective, we aim to examine how substance use screening informs referral to behavioral health treatment. Because the current sample included both a self-report measure of problem use and drug testing, aim three will compare these screening tools in relation to which YILS are referred for treatment. This study is descriptive in its aims; however, we hypothesized that: (a) more rural counties would administer fewer drug tests than less rural counties, (b) youth who had a substance use charge would receive more substance use screens than youth without a substance use charge, and (c) both screening tools would be related to behavioral health referral.

## Method

### Sample

Data were derived from the Alliances to Disseminate Addiction Prevention and Treatment project, which aimed to improve accessibility and use of evidence-based substance use treatment among legally involved youth. The research project was approved by the first authors’ Institutional Review Board (Protocol #1910282231). For the eight participating counties, the project gathered county-level administrative records from all youth who were referred to the legal system. Legal system administrative records were gathered from 1/1/2019 (i.e., the start of available data) through six months prior to the end of data collection by county to allow adequate time for a behavioral referral to be recorded in administrative data records. Seven counties ended their data collection on 11/30/2023, whereas the eighth county ended collection on 5/31/2023. Data from youth with at least one arrest after 1/1/2019 (*n* = 6,156) were initially identified. Data were then restricted to youth aged 11–17 for an initial eligible sample of *n* = 5,769. Dataset construction was at the youth level.

As part of research study procedures, counties were encouraged to implement systematic substance use screening. The research team provided training and technical assistance in evidence-based screening tools and techniques but did not require any particular instrument. All participating counties selected the CRAFFT and implemented it throughout the study. The CRAFFT is a self-report substance use screen that measures problem use and consists of six items which correspond to each letter in the acronym CRAFFT (*C*ar, *R*elax, *A*lone, *F*orget, *F*amily/friends, and *T*rouble; e.g., “I use drugs or alcohol to *R*elax.”). To examine screening during the period in which youth had the opportunity to be screened with the CRAFFT, we further restricted the sample to ensure case referral dates occurred after the implementation of the CRAFFT screen by county (i.e., as measured by the first recorded CRAFFT screening date by county). Because counties did not launch or administer the CRAFFT on standardized timelines, some counties had data with shorter time frames (implementation start dates ranged from 4/2021 to 1/2023). A total of 4,572 youth were excluded as their legal system referral occurred prior to implementation of the CRAFFT in their county, resulting in a final analytic sample of 1,197 youth.

### Measures

The following variables were extracted or constructed from administrative data records.

### Demographics

Demographics included age at time of arrest or referral, race, ethnicity, county of arrest or referral, and sex assigned at birth or gender as recorded in administrative data. Race, ethnicity, and sex or gender likely include measurement error, as data collection practices vary within and across jurisdictions. Youth demographics may be entered or reentered by law enforcement officials, probation intake staff, supervising probation officer, detention center staff, or administrative assistants. Individuals may enter the data based on youth or guardian/caregiver oral report, personal observation, or review of documents (e.g., driver’s license, past legal history). In some counties, youth are permitted to complete electronic forms that import demographic information into the legal record. The administrative data contain no information regarding the mode of data collection/entry per jurisdiction or per individual. Given the inability to differentiate sex assigned at birth and gender in the current data, we subsequently use the term “sex/gender.” Based on the 2010 Index of Relative Rurality, counties were dichotomized by rurality [22]. The Index of Relative Rurality is a continuous measure of rurality, such that greater scores indicate greater rurality. In attempts to separate the counties into two even groups and based on the distribution of scores of the participating counties (range = 0.38–0.54), if scores were less than 0.45 (the mean of the range), counties were coded as less rural (0). If scores were equal to or above 0.45, counties were coded as more rural (1). Three counties were coded more rural (range 0.45–0.54), and five were coded as less rural (range 0.38–0.42).

### Charge type

The charge for each arrest was collected. We determined the most severe charge across all arrests per youth using the following categories in descending order of severity: felony person, felony weapon, felony weapon, felony drug, felony property, felony other, misdemeanor person, misdemeanor weapon, misdemeanor drug, misdemeanor property, misdemeanor other, status offense, and other. Categorization of variables was predetermined by the administrative data collection software for jurisdictions and, thus, was consistent across counties. We were limited to the most severe change (as compared to all charges) as one participating county only recorded most severe charge. We further dichotomized the most severe charge across arrests as being related to substance use (1) or non-substance use related (0).

### Self-reported substance use and drug testing

### CRAFFT

The CRAFFT is a six-item, self-reported substance use screen measuring problem use. Example items include, “Have you ever ridden in a car driven by someone (including yourself) who has “high” or had been using drugs or alcohol?” and “Do you ever forget things you did while using drugs or alcohol?” If youth endorse two or more items, they are considered at risk for SUD, based on validation with DSM-5 criteria among youth 12–17 years old [23]. The CRAFFT demonstrated concurrent validity with substance use problems and SUD among an adolescent outpatient sample [24]. The CRAFFT is commonly used in youth legal settings, has been validated among youth residing in correctional facilities with adequate internal consistency (α = 0.85) and reliability (*r* = 0.98) [25], is predictive of future SUD among 12–17 year olds (e.g., sensitivity and specificity of 0.77 and 0.93, respectively, at two years post-baseline for moderate-severe SUD) [26], and may be more sensitive at identifying problematic substance use (in particular cannabis use) compared to other screens [14]. While the CRAFFT can be administered electronically, probation officers verbally administered the CRAFFT in the participating counties. As noted, the decision to implement the CRAFFT specifically was determined by the counties; many counties were familiar with the CRAFFT through previous administration and/or discussion with fellow counties and appreciated the length and usability, all of which facilitated uptake.

Two variables were created based on the CRAFFT screening data entered by youth legal system administrators into records: (1) a dichotomous variable indicating whether youth had any record of a CRAFFT screen (no = 0, yes = 1) and (2) a dichotomous variable indicating whether youth had any record of a positive CRAFFT screen (i.e., score ≥2; no = 0, yes = 1) over the study period. The date of the CRAFFT was also recorded.

### Drug test

Each county had different protocols for drug testing and different testing mechanisms including self-report, breathalyzer, urine, oral swab, and blood testing. The following variables were created based on administrative records of drug testing results: (1) the total number of drug tests per youth during the study period; (2) the number of positive drug tests per youth (number of tests with at least one positive drug result); and (3) dichotomous variables for whether a drug test returned positive at any point during the study period for each of the following categories of drugs: alcohol, amphetamine, anticonvulsant, barbiturate, benzodiazepine, cannabinoid, synthetic cannabinoid, cocaine, cough medicine, fentanyl, hallucinogen, inhalant, medication for opioid use disorder, methamphetamine, nicotine, opiate, and other. Screening results could either be positive (drug detected), negative (no drug detected), invalid, or unknown. Positive screens with a verified medical prescription were recoded as negative. The date of the first recorded drug test and first positive drug test after legal system referral was used to capture the ideal process of identifying youth as early as possible and to allow adequate follow-up time to examine its association with legal system referrals (as opposed to using most recent screen).

### Referral to behavioral health and substance use services

Each county documented whether youth were referred by the court or probation to complete substance use services. Two independent coders reviewed all recorded court-ordered requirements and coded all possible behavioral health services, and specifically all substance use services. In the initial round of coding, interrater reliability was moderate (κ = 0.50). Disagreements pertained to psychiatric evaluations, anger management classes, and residential placements. Coders decided to count such services as behavioral health. The second round of coding yielded improved agreement (κ = 0.70). Disagreements primarily surrounded day reporting and whether specific facilities offered behavioral health services. Finally, coders met to resolve any disagreements, including the decision to exclude day reporting, to ensure 100% agreement. In this meeting, coders also determined substance use specific services through discussion to meet 100% agreement. Substance use services were primarily identified with key words (e.g., “alcohol drug education class,” “cannabis prevention class,” “substance abuse evaluation”). Two variables were then developed: (1) behavioral health services court-ordered associated with the referent referral (any = 1, none = 0), and (2) substance use services court-ordered associated with the referent referral (any = 1, none = 0). Note that all substance use services were coded as behavioral health services. Examples of behavioral health services included psychoeducational classes, assessment, case management, and residential placement. Examples of substance use services included education classes, recovery coaching, individual therapy, and intensive outpatient treatment. The date of the service referral was also recorded. Because we were interested in the association between screening and referral to treatment, we did not include behavioral health service referrals if they occurred prior to the first screening date on record. We included both behavioral health and substance use services because limiting the analyses to substance use services only may have been overly restrictive. In many cases, it could not be determined whether court-ordered requirements in the referral information was specific to substance use services. Therefore, we wanted to examine referrals to behavioral health services broadly given the potential measurement error inherent in our definition of substance use services. Additionally, due to the comorbidity between substance use and other mental health conditions, the court-ordered requirements may not specify that substance use services were required.

### Availability of data and materials

The datasets used and/or analyzed during the current study are available from the corresponding author on reasonable request.

### Statistical analysis

Descriptive analyses were conducted to determine prevalence of screening administration, positive drug screens, and types of positive drug tests (i.e., THC, opioids), as well as the percentage of youth receiving screening types by county (Aim 1). For nominal and ordinal variables, we conducted chi-square tests between demographics variables and whether youth had received a drug test, received a CRAFFT screen, tested positive on a drug test, and tested positive on the CRAFFT screen. For ratio variables, we used independent samples *t*-test (Aim 2).

We performed logistic regression modeling to determine the association between positive screen and referral to services (Aim 3). We conducted three sets of models, each with two outcomes, for a total of six models. For model 1, a positive drug test was the primary independent variable. For model 2, a positive CRAFFT screen was the primary independent variable. For model 3, a positive drug test and a positive CRAFFT screen were both included as independent variables. To compare across models, we reported the concordance (*c*) statistic, which is equivalent to the area under the receiver operating curve for dichotomous outcomes. The receiver operating curve plots the true positive rate against the false positive rate. The *c*-statistic can be interpreted as the probability that among two individuals, the model will predict greater risk among the patient who experienced an outcome (i.e., behavioral health referral) compared to one who did not experience the outcome. The *c*-statistic ranges from 0.5 (random chance concordance) to 1 (perfect concordance). Note that we did not adjust for multiple comparisons as the sample size was not large enough for several categories, and Type II error held equal importance as Type I error as insights were intended to guide further analytic inventions rather than direct practice.

For the logistic regression models, only instances where CRAFFT screens and drug tests were administered prior to referral were included. Of the 694 youth who received a CRAFFT screen, 261 did so before the recorded behavioral health referral date. Of the 320 youth who received a drug test, 189 did so before the behavioral health referral date. To adjust for potential confounding due to demographic variables, the following fixed effects were included: race/ethnicity (dichotomized into non-Hispanic White and racial/ethnic minority), age at first arrest (dichotomized into 11–14 and 15–17 years old), sex/gender (male vs. female), and county. To improve model convergence, race and ethnicity were collapsed into one variable and dichotomized, and age at first arrest was dichotomized. To address quasi-complete separation in logistic regression models, a count of 1 was added to all cell counts for dichotomous predictors when a cell contained zero; and to further aid convergence, the Firth method was specified. All tests were two-sided at 0.05 significance, and all analyses were conducted in SAS 9.4 [27].

## Results

The sample represented 1,197 youth across eight counties. The majority of youth were male sex/gender (65.7%), White (70.6%), and non-Hispanic (61.2%). Approximately two-thirds (67.4%) of the sample were between the ages of 15 and 17 years old. Average age at first arrest was 15 years old (*SD* = 1.6 years). Most youth had only one arrest and referral to probation during the study period (79.2%); 13.3% had two cases and 7.5% had three or more. The maximum number of referrals during the study period was nine. In order of highest prevalence, the most severe charge across all charges for a youth was status offense (27.8%), misdemeanor drugs (15.4%), and misdemeanor person (15.1%). Approximately 23% of youth’s most severe charge was substance use related. Demographic variable distribution is shown in Table 1.

**Table 1:** Demographic information of the analytic sample of unique youth referral to the legal system

	*N* (%)
Sex/Gender^a^	
Female	411 (34.3)
Male	786 (65.7)
Race	
American Indian	2 (0.2)
Asian	6 (0.5)
Black or African American	229 (19.1)
Multiracial	63 (5.3)
Other	15 (1.6)
Unknown	37 (3.1)
White	845 (70.6)
Age at First Arrest	
11	20 (1.7)
12	62 (5.2)
13	119 (9.9)
14	189 (15.8)
15	283 (23.6)
16	272 (22.7)
17	252 (21.1)
Ethnicity	
Hispanic	145 (12.1)
Non-Hispanic	732 (61.2)
Unknown	320 (26.7)
Total Case Referrals Over Study Period	
1	948 (79.2)
2	159 (13.3)
3	52 (4.3)
4	21 (1.8)
>4	17 (1.4)
Most Severe Charge Over Study Period	
Felony Person	89 (7.4)
Felony Weapons	5 (0.4)
Felony Drugs	12 (1.0)
Felony Property	45 (3.8)
Felony Other	6 (0.5)
Misdemeanor Person	181 (15.1)
Misdemeanor Weapons	22 (1.8)
Misdemeanor Drugs	184 (15.4)
Misdemeanor Property	146 (12.2)
Misdemeanor Other	51 (4.3)
Status Offense	333 (27.8)
Other	123 (10.3)
Most Severe Charge is Substance Use Related	277 (23.1)

Based on 1197 unique individuals. Percentages are rounded to the nearest tenth and may not equal 100. ^a^ Sex assigned at birth and gender could not be differentiated in the current study. See main text for more details

### Aim 1: frequency of screening administration and positive screen results

Regarding frequency of substance use screens, 64.5% of the total sample received either a CRAFFT screen, drug test, or both. Prevalence of administration varied by type of screener, such that 26.7% of youth received any drug test, 58% received a CRAFFT screen, and 20.2% received both during the study period. A total of 292 youth (24.4% of total sample) screened positive on the CRAFFT, which is approximately 42% of youth who received a CRAFFT. Most youth only received the CRAFFT once, yet the maximum number of CRAFFT administrations per youth was three.

Of the 320 youth who completed a drug test, 174 (54.4%) screened positive. While we coded for a variety of substances, only alcohol, amphetamine, cannabinoid, cocaine, methamphetamine, nicotine, and opiates were detected in the study period. Cannabinoids were the most prevalent substance detected (84.5%), followed by alcohol (28.2%) and nicotine (10.3%). The average number of positive drug tests was 2.4, with a maximum number of 31. Table 2 presents frequency of screens.

**Table 2:** Frequency of substance use screens and results

	*N* (%)^a^
Ever Received Drug Test Over Study Period	320 (26.7)^a^
Ever Received CRAFFT Screen Over Study Period	694 (58.0)^a^
Ever Received Drug Test and CRAFFT Screen Over Study Period	242 (20.2)^a^
CRAFFT Results	
Positive	292 (42.1)^b^
Negative	402 (57.9)^b^
Positive Drug Screen	174 (14.5)^a^
Alcohol	49 (28.2)^c^
Amphetamine	6 (3.5)^c^
Cannabinoid	147 (84.5)^c^
Cocaine	2 (1.2)^c^
Methamphetamine	1 (0.6)^c^
Nicotine	18 (10.3)^c^
Opiate	3 (1.7)^c^
	Mean; Median; Range
Number of CRAFFT Screens	1.1; 1; 1–3
Number of Drug Tests	3.5; 1; 1–55
Number of Positive Drug Tests	2.4; 1; 1–31

^a^Based on 1197 unique youth. ^b^ Based on 694 unique youth. ^c^ Based on 174 unique youth

Percentages are rounded to the nearest tenth and may not equal 100

Counties demonstrated variability of administration of substance use screening. The percentage of youth who received a CRAFFT screen ranged from 0% to 67% across counties, and the percentage of youth who received a drug test ranged from 20.8% to 73.6%. The percentage of youth who received both types of substance use screens ranged from 0% to 54.3% by county. Supplemental Material Table 1 presents the screening percentage by county.

### Aim 2: screening differences across demographic variables

#### Drug test administration

Chi-square tests revealed no significant differences in drug test administration by sex/gender or race. Differences in administration of a drug test differed by ethnicity, most severe charge relating to substance use, and rurality. Non-Hispanic youth received fewer drug tests compared to Hispanic and unknown ethnicity youth (χ^2^(2) = 64.55, *p* < 0.001); youth whose most severe charge was substance use related received more drug tests than those whose charge was not substance use related (χ^2^(1) = 8.61, *p* = 0.003); and youth living in more rural counties received fewer drug tests compared to those in less rural counties (χ^2^(1) = 57.08, *p* = < 0.001). *T*-tests indicated no significant differences by age at first arrest and administration of drug test but did reveal a significant difference by number of referrals. Youth who had a drug screen had more referrals over the study period compared to youth without a drug screen (*t*(1195) = -7.03, *p* < 0.001).

#### CRAFFT screen administration

CRAFFT administration did not differ by race and ethnicity. Sex/gender, most severe charged related to substance use, and rurality were associated with differences in CRAFFT administration. Male youth received more CRAFFT screens than female youth (χ^2^(1) = 4.55, *p* = 0.033). Youth whose most severe charge was substance use-related received more CRAFFT screens compared to non-substance use-related severe charges (χ^2^(1) = 20.24, *p* < 0.001). Youth living in more rural counties received fewer CRAFFT screens compared to those in less rural counties (χ^2^(1) = 12.94, *p* < 0.001). *T*-tests indicated a significant difference in CRAFFT administration by age at first arrest but not number of referrals. Youth who received a CRAFFT were older than those who did not receive it (*t*(1195) = -2.71, *p* = 0.007). Table 3 presents chi-square and *t*-tests for screening administration.

**Table 3:** Chi-square tests and t-tests by screening administration and demographics

	Administration of Drug Test		
Demographic Variable	No (N, Row %)	Yes (N, Row %)	Chi-Square (DF), p-value
Sex/Gender^a^			0.01 (1), *p* = 0.904
Female	302 (73.5)	109 (26.5)	
Male	575 (73.2)	211 (26.8)	
Race			1.86 (2), *p* = 0.390
White	612 (72.4)	233 (27.6)	
Black or African American	176 (76.9)	53 (23.1)	
Racial Minority or Unknown	89 (72.4)	34 (27.6)	
Ethnicity			**64.55 (2)**, ***p < 0.001***
Hispanic	91 (62.8)	54 (37.2)	
Not Hispanic	596 (81.4)	136 (18.6)	
Unknown	190 (59.4)	130 (40.6)	
Most Severe Charge is Substance Use Related			**8.61 (1)**, ***p = 0.003***
No	693 (75.3)	227 (24.7)	
Yes	184 (66.4)	93 (33.6)	
Rurality			**12.94 (1)**, ***p < 0.001***
Less rural	340 (38.9)	534 (61.1)	
More rural	163 (50.5)	160 (49.5)	
	No (N, Mean)	Yes (N, Mean)	t-Value (DF), p-value
Age at First Arrest	877 (15.0)	320 (15.2)	-1.82 (1195), *p* = 0.069
Total Number of Referrals over Study Period	877 (1.2)	320 (1.6)	**-7.03 (1195)**, ***p < 0.001***
	**Administration of CRAFFT Screen**		
	No (N, Row %)	Yes (N, Row %)	Chi-Square (DF), p-value
Sex/Gender^a^			**4.55 (1)**, ***p = 0.033***
Female	190 (46.2)	221 (53.8)	
Male	313 (39.8)	473 (60.2)	
Race			3.37 (2), *p* = 0.185
White	342 (40.5)	503 (59.5)	
Black or African American	108 (47.2)	121 (52.8)	
Racial Minority or Unknown	53 (43.1)	70 (56.9)	
Ethnicity			3.80 (2), *p* = 0.149
Hispanic	52 (35.9)	93 (64.1)	
Not Hispanic	322 (44.0)	410 (56.0)	
Unknown	129 (40.3)	191 (59.7)	
Most Severe Charge is Substance Use Related			**20.24 (1)**, ***p < 0.001***
No	419 (45.5)	501 (54.5)	
Yes	84 (30.3)	193 (69.7)	
Rurality			**57.08 (1)**, ***p < 0.001***
Less rural	589 (67.4)	285 (32.6)	
More rural	288 (89.2)	35 (10.8)	
	No (N, Mean)	Yes (N, Mean)	t-Value (DF), p-value
Age at First Arrest	503 (14.9)	694 (15.2)	**-2.71 (1195)**, ***p = 0.007***
Total Number of Referrals over Study Period	503 (1.3)	694 (1.4)	-0.92 (1195), *p* = 0.356

Based on 1197 unique individuals. Percentages are rounded to the nearest tenth and may not equal 100. Bolded indicates significant at *p* < 0.05. aSex assigned at birth and gender could not be differentiated in the current study. See main text for more details

#### Positive drug test

Positive drug test did not differ by sex/gender, race, ethnicity, and rurality. Most severe charge being substance use related, age at first arrest, and number of referrals were associated with differences in positive drug test. Youth with a positive drug test had a most severe charge that was related to substance use compared to youth with a negative test (χ^2^(1) = 9.44, *p* = 0.002). Youth who had a positive drug test score were older (*t*(318) = -2.61, *p* = 0.010) and had more referrals over the study period compared to youth who tested negative (*t*(318) = -2.20, *p* = 0.028).

#### Positive CRAFFT Screen

Positive CRAFFT screen did not differ by sex/gender or rurality. There was significant difference among positive CRAFFT screens by race and ethnicity. White youth screened positive more than Black/African American or youth from other minoritized background (χ^2^(2) = 21.55, *p* < 0.001); youth with unknown ethnicity screened positive more than Hispanic or not Hispanic ethnicity (χ^2^(2) = 37.69, *p* < 0.001). Youth who screened positive had more severe charges related to substance use compared to those who screened negative (χ^2^(1) = 112.46, *p* < 0.001). Youth who had a positive CRAFFT score were older (*t*(692) = -5.40, *p* < 0.001) and had more referrals over the study period compared to youth who screened negative (*t*(692) = -3.23, *p* = 0.001). Table 4 presents chi-square and *t*-tests for positive screen (Table 5).

**Table 4:** Chi-square tests and t-tests by positive screen and demographics

	Positive Drug Test		
Demographic Variable	No (N, Row %)	Yes (N, Row %)	Chi-Square (DF), p-value
Sex/Gender^a^			0.03 (1), *p* = 0.863
Female	49 (45.0)	60 (55.1)	
Male	97 (46.0)	114 (54.0)	
Race			0.52 (2), *p* = 0.772
White	109 (46.8)	124 (53.2)	
Black or African American	22 (41.5)	31 (58.5)	
Racial Minority or Unknown	15 (44.1)	19 (55.9)	
Ethnicity			5.97 (2), *p* = 0.051
Hispanic	31 (57.4)	23 (42.6)	
Not Hispanic	65 (47.8)	71 (52.2)	
Unknown	50 (38.5)	80 (61.5)	
Most Severe Charge is Substance Use Related			**9.44 (1)**, ***p = 0.002***
No	116 (51.1)	111 (48.9)	
Yes	30 (32.3)	63 (67.7)	
Rurality			0.14 (1), *p* = 0.711
Less rural	129 (45.3)	156 (54.7)	
More rural	17 (48.6)	18 (51.4)	
	No (N, Mean)	Yes (N, Mean)	t-Value (DF), p-value
Age at First Arrest	146 (15.0)	174 (15.4)	**-2.61 (318)**, ***p = 0.010***
Total Number of Referrals over Study Period	146 (1.5)	170 (1.7)	**-2.20 (318)**, ***p = 0.028***
	**Positive CRAFFT Screen**		
	No (N, Row %)	Yes (N, Row %)	Chi-Square (DF), p-value
Sex/Gender^a^			0.25 (1), *p* = 0.619
Female	125 (56.6)	96 (43.4)	
Male	277 (58.6)	196 (41.4)	
Race			**21.55 (2)**, ***p < 0.001***
White	265 (52.9)	238 (47.3)	
Black or African American	90 (74.4)	31 (25.6)	
Racial Minority or Unknown	47 (67.1)	23 (32.9)	
Ethnicity			**37.69 (2)**, ***p < 0.001***
Hispanic	54 (58.1)	39 (41.9)	
Not Hispanic	272 (66.3)	138 (33.7)	
Unknown	76 (39.8)	115 (60.2)	
Most Severe Charge is Substance Use Related			**112.46 (1)**, ***p < 0.001***
No	352 (70.3)	149 (29.7)	
Yes	50 (25.9)	143 (74.1)	
Rurality			2.51 (1), *p* = 0.113
Less rural	318 (59.6)	216 (40.5)	
More rural	84 (52.5)	76 (47.5)	
	No (N, Mean)	Yes (N, Mean)	t-Value (DF), p-value
Age at First Arrest	402 (14.9)	292 (15.5)	**-5.40 (692)**, ***p < 0.001***
Total Number of Referrals over Study Period	402 (1.3)	292 (1.9)	**-3.23 (692)**, ***p = 0.001***

Based on 1197 unique individuals. Percentages are rounded to the nearest tenth and may not equal 100. Bolded indicates significant at *p* < 0.05. ^a^Sex assigned at birth and gender could not be differentiated in the current study. See main text for more details

**Table 5:** Association between screening and behavioral health referral

	Behavioral Health Referral		Substance Use Referral	
	Odds Ratio (95% CI)	c-statistic	Odds Ratio (95% CI)	c-statistic
*Model 1*:				
Positive drug test	8.48 (6.06–11.87)	0.743	8.74 (6.29–12.14)	0.736
*Model 2*:				
Positive CRAFFT screen	11.55 (8.33–16.01)	0.771	10.40 (7.55–14.31)	0.762
*Model 3*:				
Positive drug test	3.22 (2.43–4.26)	0.776	3.59 (2.71–4.77)	0.790
Positive CRAFFT screen	4.70 (3.60–6.13)		4.53 (3.45–5.95)	

Fixed effects included for race/ethnicity, age, sex/gender, and county

### Aim 3: association with referral

Positive substance use screens were positively associated with behavioral health and substance use referral across all models. In model 1, having a positive drug test prior to referral was associated with increased odds of behavioral health referral (Odds Ratio [OR] = 8.48, 95% Confidence Interval [CI] [6.06–11.87]) and substance use referral (OR = 8.74, 95% CI [6.29–12.14]). A similar pattern was observed in model 2, albeit with greater magnitude, between a positive CRAFFT screen and behavioral health referral (OR = 11.55, 95% CI [8.33–16.01]) and substance use referral (OR = 10.40, 95% CI [7.55–14.31]). For model 3, positive drug test (OR = 3.22, 95% CI = 2.43–4.26) and positive CRAFFT screen (OR = 4.70, 95% CI [3.60–6.13]) were associated with behavioral health referral. Positive drug test (OR = 3.59, 95% CI [2.71–4.77]) and positive CRAFFT screen (OR = 4.53, 95% CI [3.45–5.95]) were also associated with substance use referral. *C*-statistics were comparable across models, however, were lowest for model 1 and highest for model 3, specifically when examining substance use referral (*c* = 0.790).

## Discussion

The aim of the current paper was to investigate the patterns of two types of substance use screens (self-report CRAFFT and drug tests) across eight Indiana counties, examine differences in screening by demographic variables, and examine the association between screening positive and court-ordered referral to either behavioral health or substance use services. We limited the scope of the cohort to include youth who were referred to county probation offices after the implementation of the CRAFFT screen. Therefore, the current investigation offers descriptive insight into substance use screening patterns at the implementation onset of a screening measure, which may or not be concurrent to drug testing depending on the county.

### Aim 1: frequency of screening administration and positive screen results

The results demonstrated that CRAFFT screens were more frequently administered than drug tests. While only approximately a quarter of youth received a drug test, over half (58%) received a CRAFFT screen at some point during the study period. Two enrolled counties accounted for most drug tests; these counties tested about two-thirds of their youth and were both considered less rural. Chi-square tests revealed that youth in more rural counties received fewer drug tests and CRAFFT screens than those in less rural counties. While the range of rurality scores was limited in the current study, lower screening prevalences in more rural counties is consistent with prior literature. Using survey data among 195 community supervision agencies in 20 states, Marks and colleagues (2019) documented that rural agencies used fewer screening measures with alcohol, tobacco, prescription and illicit drug items, as well as fewer drug tests. The CRAFFT was frequently used among rural agencies, only second to the Massachusetts Youth Screening Instrument-Version 2 (MAYSI-2) [20]. While the results from the current study cannot clarify the facilitators and barriers to CRAFFT implementation compared to drug tests, these results may speak to the limited feasibility of drug tests and greater feasibility and acceptability of self-report measures among more rural counties based on resource constraints. Notably, while our research team encouraged adoption of any evidence-based youth substance use screen, all counties selected the CRAFFT, which may attest to the perceived acceptability, feasibility, and/or utility of the measure. In particular, the CRAFFT may be optimal to detect problematic cannabis use compared to the MAYSI-2 [14]. Staff perceptions of screener utility in relation to specific substances would be beneficial to investigate in future studies.

The study highlighted county-level variability in screener utilization. As reported above, drug tests predominantly occurred in less rural counties. CRAFFT implementation dates varied by county, as well as the percentage of youth who were screened with the CRAFFT (ranging from 20% to 73%). We did not examine whether CRAFFT screening prevalence changed over time. Future studies would benefit from examining trends in screening coverage since the onset of screener implementation. Regardless, screening was not universal.

### Aim 2: screening differences across demographic variables

Drug test administration differed by ethnicity and CRAFFT administration differed by sex/gender; non-Hispanic youth received fewer drug tests and female youth received fewer CRAFFT screens. While not specific to screening administration, prior research has documented that Black and Latinx YILS had fewer referrals to substance use services than White YILS [21, 28], and youth in rural counties were referred less frequently than those in urban counties [29]. More generally, prior research has found that individuals belonging to non-minoritized racial and ethnic groups are least likely to have more restrictive legal system involvement [1], and that rural areas (which administer fewer drug tests) are less likely to serve Hispanic and Black youth [20]. Therefore, study results may be a result of various factors at the county-level, such as differences in youth served, how drug tests are used (e.g., first screening versus conditions of probation), and substance(s) endorsed or detected (which was not explored in the current study). Additionally, systemic factors may be at play in explaining group differences, such as implicit biases of personnel in the youth legal system and systemic racism that place minoritized youth at continued disadvantage compared to White youth. To better understand how systemic processes contribute to inequitable outcomes, research-community partnerships are needed to further investigate and develop strategies to promote equitable screening (and referral).

More probation referrals over the study period were associated with drug test administration and older age was associated with CRAFFT administration. Most severe charge being related to substance use was associated with administration of drug test and CRAFFT. These results likely cluster together in demonstrating that youth who are more frequently in contact with the legal system have more opportunity for screening administration and alleged substance use is used to indicate targeted substance use screening. This, in combination with non-universal screening results in Aim 1, suggest that there is likely a missed opportunity to screen YILS for substance use during their contact with the legal system. Universal screening may indicate need for services beyond alleged charge and serve to prevent future risk for severe substance use and legal involvement. Incorporating such universal screening should be informed by implementation science frameworks (whether in practice or in research).

Our descriptive analyses examining group differences by screening administration may offer initial insight into who receives a drug test or CRAFFT screen. However, ongoing research is needed to understand the individual and agency-level barriers and facilitators into screening adoption. For example, it is possible that while screening may be perceived as acceptable, agency staff may be uncertain of how to proceed after a screen is positive and/or may be unaware of or lack confidence in the (often limited) local behavioral health resources. At the agency level, prior literature found an association between perception of substance use need within the population an agency is serving and provision of prevention services [30]. Interventions, such as Family Connect, have targeted the linkage gap between probation settings and behavioral health treatment [31] and may offer a promising venue to improve the administration and actionability of substance use screens among community supervised youth.

Regarding positive results, more legal involvement, substance use charges, and older age of first arrest were all associated with positive drug tests or CRAFFT screens. These results call upon the importance of early identification and intervention to prevent further legal involvement and problem substance use. Additionally, positive CRAFFT screens differed by race and ethnicity. Given small cell sizes among racial minorities, as well as a large portion of unknown ethnicity, our ascertainment of race and ethnicity was crude. Increased sample sizes to investigate within racial and ethnic group are greatly needed to further understand racial and ethnic differences on positive CRAFFT screens.

### Aim 3: association with referral

When examining the association between screen type and treatment referral, a positive CRAFFT score outperformed a positive drug test as defined by the *c*-statistic. However, when both screen types were analyzed in the model, the *c*-statistic was highest in association with substance use referral. A combination of positive drug tests and CRAFFT screens appear to be most informative when understanding which youth are referred to treatment based on substance use screening information. When considered alone, the CRAFFT screen had a higher *c*-statistic compared to the drug test. Therefore, probation officers and agency staff may be utilizing information from the CRAFFT screen to make referral recommendations differently than drug test results. Prior research has demonstrated that individual-level factors accounted for more variance than county-level factors in predicting court referral rates, such that those identified to be in need were more likely to be referred to substance use treatment [29]. However, we are unaware of research examining referral by the specific indicator of substance use (drug test versus self-reported screen). We also suggest that future research examine moderators (e.g., race and ethnicity) between screening and behavioral health referral. Examining whether the relation between screening type and treatment referral varies by demographic factors, such as race, may elucidate disparities that can inform the implementation of tailored interventions.

### Practice and research implications

At face value, the CRAFFT has items to identify problem substance use, whereas a drug test does not necessarily do so. In fact, one study identifying latent classes among a sample of 210 YILS in rural counties found that 70% of youth were in the lowest risk group in terms of offending and serious substance use [32]. Therefore, screening measures that identify problem use may be useful in making behavioral health referrals, especially among youth who are new to the legal system. The CRAFFT, or other self-report measures, may be particularly actionable upon initial screen compared to drug testing, which may require additional time to wait for results. Considering the utility of drug tests versus CRAFFT screens also raises considerations about identifying versus monitoring substance use. Consistent with the range of number of drug tests (1–55) versus CRAFFT screens (1–3) by youth in the current sample, drug tests were likely used to monitor ongoing use relevant to formal or informal probation requirements. Especially for less resourced counties that minimally administer or do not administer drug tests, screens that capture problem substance use may be particularly helpful in triaging youth to other services that may routinely monitor use. Especially given the low availability and access to youth substance use disorder treatment, probation offices may want to provide evidence-based services within their probation agency. Task-shifting, such as with contingency management interventions, is a logical next step after the use of the CRAFFT to intervene with youth at highest risk of problem substance use [33].

Uptake of screening and subsequent referral to behavioral health treatment has been a focus of implementation science with multi-site randomized controlled trials investigating the impact of system-level interventions. JJ-TRIALS, for example, found that counties trained in conducting quality improvement had greater YILS service utilization than control counties. While the main results focused on connection to/use of care rather than screening, the results offer insights into intervention options focused at the system level (e.g., legal, mental health) [34]. Other interventions, such as Stepping Up, guide counties to commit to, develop, and implement an action plan to address gaps in behavioral health services among legal-involved adults; Stepping Up requires counties to adopt a universal screening tool [35]. Together, existing trials informed by implementation science may measure uptake of screening or stipulate its use. However, research is critically needed to help inform what conditions and strategies aid counties to increase screening administration.

### Strengths and limitations

The study was strengthened by the use of youth-level administrative data in multiple counties within one state to examine the variability across counties in substance use screening practices. As mentioned, all counties adopted the CRAFFT screener, which offered the opportunity to compare different types of screening measures. The CRAFFT is efficient, easy to use, and has a validated cutoff.

The findings should be considered within the context of numerous limitations. First, drug tests do not denote a complete picture of actual or persistent substance use. While it may be optimal to utilize both drug tests and self-report screens repeatedly throughout legal involvement, this is often not feasible for probation agencies. Therefore, identifying when substance use screens are used to aid standardized recommendations may be beneficial in future studies. For example, Brusman Lovins and colleagues (2024) examined these questions among a predominately adult sample. Second, we lacked information on whether youth were concurrently connected to behavioral health services. When examining referral, absence of referral may indicate no referral was made, but may also indicate that youth were already in services. Therefore, our aim was not to increase the *c*-statistic and explain who was or was not referred. Rather, we aimed to compare drug tests and the CRAFFT in understanding referral. Related, we only compared model performance using one metric (*c*-statistic) rather than other model performance statistics. Third, numerous counties included drug tests that were classified as “admission,” in which youth admitted their use. In these instances, it is unclear whether a drug test was administered in conjunction with admitted use, or admitted use replaced a drug test. Fourth, we used a measure of rurality that crudely dichotomized counties. Studies may benefit from considering a variety of county measures, such as overdose rate. Fifth, we did not conduct mixed effects modeling to account for clustering of observations by county due to model convergence issues. While we accounted for county as a fixed effect, a more statistically rigorous analysis to account for clustering was not possible in the current study.

## Conclusions

In eight Indiana counties after the implementation of the CRAFFT screener, we descriptively examined patterns of screening administration and positive screen, demographic differences among administration and positive screen, and association with treatment referral. Results indicated more youth were screened with the CRAFFT than drug tests, substantial county-level variability in the percentage of youth who received substance use screening, and significant associations between positive drug tests or CRAFFT screen and referral. When understanding which youth are referred to treatment, positive CRAFFT scores may be more informative than positive drug tests. Future research is needed to better identify when youth should receive screening and what type of screening is optimal, which may help identify screening recommendations for probation offices. Regardless of improvements in screening, more research is needed to link youth to behavioral health resources post-screening.

## Supplementary Information

Below is the link to the electronic supplementary material.

Supplementary Material 1

## Data Availability

The datasets used and/or analyzed during the current study are available from the corresponding author on reasonable request.

## Abbreviations

SUD: Substance use disorder
YILS: Youth involved in the legal system
CRAFFT: Car, Relax, Alone, Forget, Family/friends, and Trouble
OR: Odds ratio
CI: Confidence interval
MAYSI-2: Massachusetts Youth Screening Instrument-Version 2
