# Supplementary material for: A descriptive analysis of substance use screening among youth involved in the legal system in eight counties
Source: Addict Sci Clin Pract. 2025 Oct 21;20:84. doi: 10.1186/s13722-025-00609-3 (PMC12542231; doi:10.1186/s13722-025-00609-3)
Supplement: Supplementary file 1 — Supplementary Material 1 [file 13722_2025_609_MOESM1_ESM.docx]

| Supplemental Material Table 1. Percentage of screening completion by county. | | | |
| --- | --- | --- | --- |
|  | Percentage of Youth who Received Drug Tests | Percentage of Youth who Received CRAFFT Screening | Percentage of Youth who Received Both Drug Tests and CRAFFT Screening |
| County 1 | 13.9 | 52.0 | 11.1 |
| County 2 | 1.7 | 51.7 | 0 |
| County 3 | 14.7 | 63.8 | 11.4 |
| County 4 | 67.0 | 25.8 | 24.7 |
| County 5 | 15.0 | 66.7 | 15.0 |
| County 6 | 60.4 | 73.6 | 54.3 |
| County 7 | 0 | 50.9 | 0 |
| County 8 | 4.2 | 20.8 | 0 |
| Percentages are calculated by county (i.e., youth in that county that have received screen(s) during the study period). Youth may have received none, one, or both screeners; groups are not mutually exclusive. | | | |
